# Similarity and Dissimilarity in Perceptual Organization: On the Complexity of the Gestalt Principle of Similarity

**DOI:** 10.3390/vision6030039

**Published:** 2022-06-28

**Authors:** Baingio Pinna, Daniele Porcheddu, Jurgis Skilters

**Affiliations:** 1 Department of Biomedical Sciences, University of Sassari, 07100 Sassari, Italy; 2 Department of Economics and Business, University of Sassari, 07100 Sassari, Italy; daniele@uniss.it; 3 Department of Computer Science, University of Latvia, LV-1586 Latvia, Latvia; jurgis.skilters@lu.lv

**Keywords:** shape perception, perceptual organization, Gestalt principle of similarity, principle of accentuation, visual illusion

## Abstract

The main purpose of this work is to explore the Gestalt principle of similarity and to demonstrate that the use of this term alone is not sufficient to understand the dynamics of grouping fully and correctly. More generally, this work aims to show that the Gestalt principle of similarity alone is not sufficient for a full understanding of perceptual organization occurring both in the classical and mostly in the new phenomena here presented. Limits and incompleteness of the similarity principle have suggested the basic, more general and stronger role of dissimilarity in perceptual grouping under a large variety of conditions. Dissimilarity was shown as a basic principle of figure–ground segregation, as a tool useful to create at will new groups and visual objects within patterns where they are totally invisible, as an attribute that is able to accentuate different shape components within the same object, as a way to distort shapes and create visual illusions, but also to reduce or annul them and, finally, to decompose, ungroup and reshape single objects. The results demonstrated the necessity to introduce a principle of dissimilarity that is complementary to similarity as already studied by Gestalt psychologists.

## 1. The Gestalt Principle of Similarity

**Figure 1:**
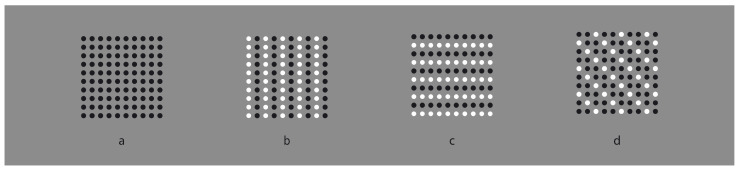
The Gestalt principles of proximity and similarity: all else being equal, the closest and most-similar elements are grouped together creating an uniform square matrix (**a**), columns (**b**), rows (**c**) and oblique arrangements (**d**).

In Figure 1a, an array of black dots is illustrated. Starting from simple stimuli such as this, Wertheimer [1,2,3,4] first explored the general problem of grouping and perceptual organization. He answered the following, still-relevant questions—how do individual elements in the visual field “go together” to form an integrated percept? How do they create wholes?—by discovering the following well-known principles: proximity, similarity, good continuation, closure, symmetry, convexity, Prägnanz, past experience, common fate and parallelism. In particular, the proximity principle states that, all else being equal, the closest elements are grouped together.

In Figure 1a, the proximity groups the dots along the vertical and horizontal directions, creating a square matrix of rows and columns. Diagonal arrangements are very dissimilar and are difficult to perceive in spite of attentional efforts. It is worth noting that the similarity/equality of proximities between rows and columns makes the perception very reversible, switching rows to columns and vice versa. None of the two directions win; however, their similarity/equality in terms of proximity imparts to the square matrix a strong sense of stability and equilibrium between the two directions. Easily, it follows that the salience of rows or columns can be alternatively enhanced or weakened by making the distance among them dissimilar (not illustrated).

However, to break this stability, without playing with the proximity principle, it is sufficient to introduce another kind of dissimilarity among columns and rows, as illustrated, respectively, in Figure 1b,c. By reversing the contrast, the salience of the two orthogonal directions is now much stronger than the one of Figure 1a. Stated differently, the similar proximities of rows and columns can be broken by introducing dissimilarities in between them, for example, a kind of similarity different from proximity. Analogous outcomes can be obtained by making the color, the shape or other attributes of the elements dissimilar. Most of the stimuli here presented are based on contrast polarity (black and white elements within a 50% gray background) since it likely elicits the strongest dissimilarity effect among elements [5,6], given the greatest luminance difference.

In the same way as before, diagonal grouping of dots can be highlighted through the contrast polarity as shown in Figure 1d. Under this condition, the new kind of grouping is playing independently and against the proximity principle, since the amplitude of diagonals interspaced between elements is longer than the horizontal/vertical ones. According to Wertheimer, the similarity principle is responsible for the grouping, stating that, all else being equal, the most similar elements are grouped together creating columns, rows and oblique wholes, respectively, in Figure 1b–d.

At first sight, Wertheimer’s description appears, immediately, to be the only possible one that is also consistent with the described outcomes. As proof, it is no coincidence that the similarity principle has never been challenged before (for a unique exception, see Vicario, 1998). Some authors looked at how similarity up to a low similarity and discontinuity allowed regions to be grouped together and segmented [7,8,9,10,11,12,13].

Nevertheless, the main purpose of this work is to demonstrate that the use of this term alone is not sufficient to understand the dynamics of grouping fully and correctly and, more generally, that it is not sufficient for a full understanding of perceptual organization occurring in the previous and in the following conditions. Rather, it is necessary to introduce a complementary principle of dissimilarity. The following new phenomena will test the limits and incompleteness of the similarity principle.

## 2. Similarity and Dissimilarity

Starting from the previous phenomenal descriptions, a first immediate note suggests that *dissimilarities* by reversed contrast are strong enough to break similar/equal proximities among columns and rows and to highlight one or the other arrangement. Conversely and at the same time, contrast *similarities* among dots are responsible for putting them together in distinct wholes. In layman’s terms, dissimilarities could be responsible for creating segregation, distinction and separation between adjacent groups and similarities for putting the dots within each group together homogeneously.

Under these simple conditions, dissimilarities and similarities seem to interact synergistically, appearing in different groups, highlighting the differences among groups and the homogeneities within each group. It follows that a complete definition of a whole should contain at least two complementary dynamics: dissimilar interactions, such as dividing boundaries aimed at emphasizing discontinuities among regions of the visual field, and similarities to enhance the inner homogeneity and, thus, to contribute to the improvement of the salience of the segregated object. In other terms, dissimilarities highlight and segregate the boundaries of an object and similarities highlight and segregate its inner surface and matter. These preliminary remarks could be tested through the following stimuli.

**Figure 2:**
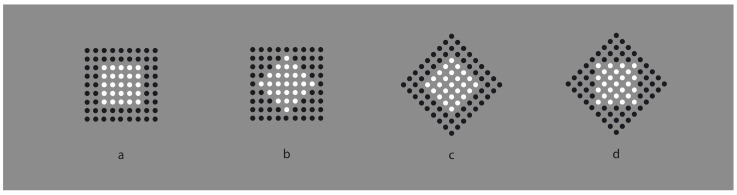
Homogeneous squares (**a**,**d**) and diamonds (**b**,**c**) clearly segregated from surrounding homogeneous black dots.

In Figure 2, the similarity principle seems to create wholes more complex than the condition illustrated in Figure 1b,c, where the groups appear as juxtaposed vertical and horizontal line segments. Now, inner homogeneous surfaces are perceived as clearly segregated from surrounding homogeneous black dots. The two black and white components of each condition are not perceived as juxtaposed but as organized in terms of figure–ground segregation, where the inset white region is perceived as a surface placed in front of a full matrix of dots placed on the background. The amount of segregation between a figure and background is directly proportional to the dissimilarity along the boundaries of the white surface, i.e., between the black and white sets of dots. In fact, by reducing the contrast between the two regions, the segregation is reduced accordingly (not illustrated).

Dissimilarity clearly induces segregation. On the other hand, the inner homogeneity among the elements of the adjacent regions is also related to the salience of the perceived segregation by imparting the surface/background scission. In summary, dissimilarities are related to the separation between adjacent regions, the boundary formation and the unilateral belongingness of the boundaries [14,15], while similarities define the inner surface qualities of the segregated object that further contribute to the strength of the figure–ground segregation.

These preliminary notes suggest that the Gestalt principle of similarity could operate as a figure–ground principle. However, to act as such, it would require a further principle of dissimilarity, essential in figure–ground segregation. In fact, while grouping requires putting things together, figure–ground segregation implies separation, discontinuity and, thus, dissimilarity.

**Figure 3:**
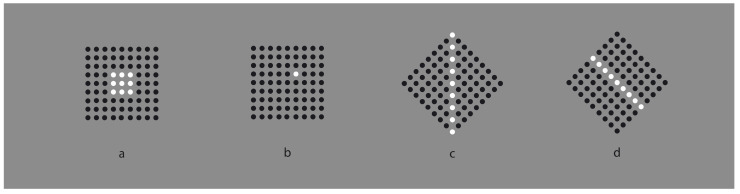
A white surface (**a**) is reduced to a single dot (**b**), to a vertical or to an oblique line segment (**c**,**d**).

This phenomenal description can explain limiting conditions such as those shown in Figure 3, where the similarity of the elements creating a surface (Figure 3a) is reduced to a single dot (Figure 3b) or to a vertical or oblique line segment (Figure 3c,d).

**Figure 4:**
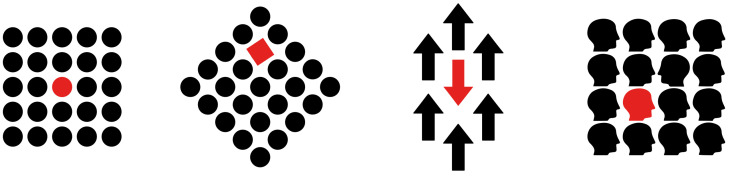
The dissimilarity reveals the red element.

It is worth noticing that the reduction to a single dot cannot be described in terms of similarity. The most appropriate term should be “dissimilarity” [16]. In fact, the most general and meaningful notion of dissimilarity occurs when only one element reveals itself as different from the others, as shown in Figure 4.

The second and third patterns of Figure 4 show two dissimilarities at the same time: shape and color, in the second pattern, and orientation and color in the third. It should be noted that the fourth pattern contains two different dissimilarities: one is related to the color; the other is related to the orientation of the profile. The most salient and, therefore, the most different and dissimilar is the chromatic one. This suggests that a dissimilarity that makes a dissimilarity is necessary to reveal new objects or, more simply, to highlight element components.

It is essential to consider that the same dissimilarity perceived in Figure 3b and Figure 4 occur also with a single shape alone on an empty background, e.g., when the surrounding black dots of Figure 4 are removed, leaving the red circle alone on the white background (not illustrated). Again, we can state that a single red circle placed on a homogeneous background appears as a figure because of the dissimilarity, discontinuity and inhomogeneity in relation to the surrounding homogeneous visual field. The term similarity can be used only to describe the inner surface of the circle. The notion of dissimilarity seems to be more general and useful for the figure–ground segregation than for similarity. In other words, dissimilarity does not need similarity to deliver segregated objects, although similarity can support its role by defining the inner matter of the object.

It should be mentioned that, in Figure 3c,d, the two diamond wholes are divided in two by the vertical and oblique arrangement of white dots. However, they belong to the wholes; they are part of them and emerge as their central boundaries. The dissimilar arrangements are, under these conditions, segregated and, at the same time, part of the wholes. In the light of this observation, the double complementary dynamics of dissimilarity and similarity appear even more necessary. In fact, only by taking into account both notions is it possible to describe appropriately the belongingness of the arrangements to the wholes, even if they are dissimilar.

There is a further intriguing observation that requires description. Upon closer inspection of Figure 3c,d, the vertical and oblique arrangements reorient their wholes along the directional symmetry they created. As a consequence, the geometrical diamond-like shapes of the wholes are, respectively, perceived as a diamond in Figure 3c and as a rotated square in Figure 3d. Let us postpone the discussion on this important point until further and more effective examples are presented.

Moreover, the two arrangements reveal two axes of symmetry of their wholes, in the same way the single white dot of Figure 3b clearly appears not at the center of the square matrix. However, it shows the exact position of the center [17] Single dots within wholes indicate not only their locations but also special attributes of the wholes, such as the center, angles and sides, as we shall see below.

Finally, by taking a closer look at the stimuli of Figure 2 and Figure 3b,c, we can see that the white similar/dissimilar dots strongly influence the arrangement and the grouping of the black surrounding elements, which are instead expected to be ruled by the proximity principle. However, the kind of grouping of the inset components also influences the grouping of the surrounding ones, which appear reoriented in parallel to the sides of the white diamond (e.g., Figure 2c) or scattered similarly to the white square (Figure 2d). The group leading and imparting the arrangement of the other group is the one that appears dissimilar, i.e., the inner white elements appearing as a figure segregated from the black background. More examples will be shown below.

**Figure 5:**
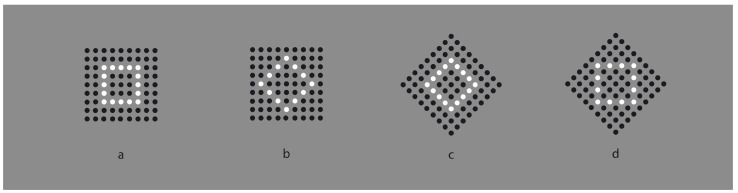
Square (**a**,**d**) and diamond (**b**,**c**) contours made up of white dots.

In Figure 5, the surfaces of Figure 2 are reduced to their contours so that the role of dissimilarity is enhanced, as is also the spreading of the kind of arrangement to the black dots all around. More particularly, the grouping of the white shapes related to the similarity and proximity induces the same kind of organization on the black dots, while the grouping of the white shapes related to the similarity and remoteness along the virtual diagonal induces the same arrangement on the black dots.

**Figure 6:**
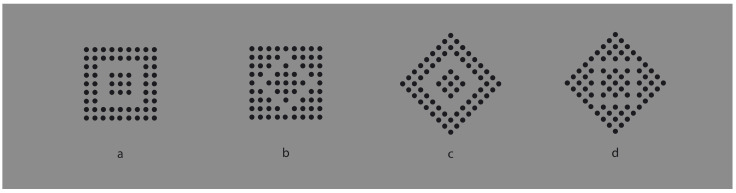
Missing squares (**a**,**d**) and diamonds (**b**,**c**).

By removing the white dots of Figure 5, the arrangement of the black dots does not change (Figure 6). In fact, the missing shapes are perceived as dissimilarities or, equivalently, as inhomogeneities. Under these conditions, it is not phenomenally correct to say that the perceived grouping is due to the similarity principle. In terms of the Gestalt principle, only the proximity should be mentioned, creating two different groups for each condition as illustrated in Figure 6. However, this prediction is not consistent with the perceived results.

**Figure 7:**
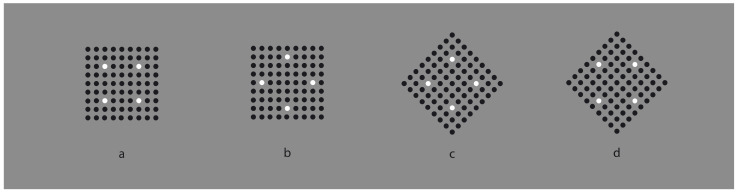
Virtual squares (**a**,**d**) and diamonds (**b**,**c**).

It is even more intriguing to obtain the same kind of grouping previously described with only four white dots (Figure 7), which can be seen as virtual squares and diamonds.

**Figure 8:**
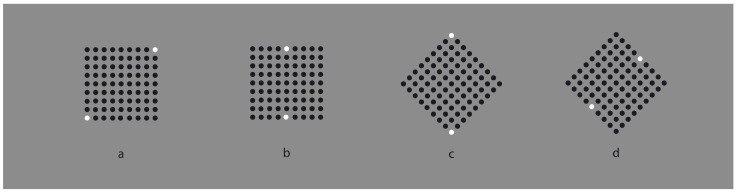
Poles for a specific virtual direction (diagonal, (**a**,**c**), and side, (**b**,**d**)).

The same effects, although weaker, are also perceived in Figure 8 with only two white dots that behave like poles or accents for a specific virtual direction [18,19,20]. Again, the notion of similarity is, here, very flimsy and useless.

**Figure 9:**
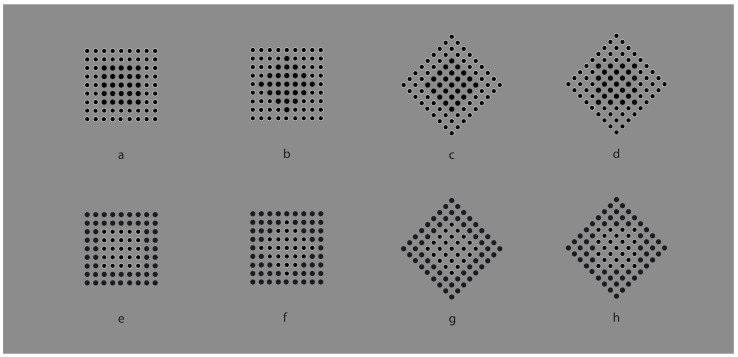
Squares (**a**,**d**,**e**,**h**) and diamonds (**b**,**c**,**f**,**g**) appearing as figures more saliently than those illustrated in Figure 2.

The basic role played by dissimilarity to segregate and accentuate the boundaries is supported by the conditions illustrated in Figure 9, where the figure–ground segregation is even stronger than in the previous conditions, although the amount of similarity is higher than the conditions with fully white dots. The dissimilarity, localized only on the boundaries of the dots, highlights figure–ground segregation and induces a prominent depth displacement. A sliding motion effect can also be perceived by randomly shaking the stimuli [21].

In summary, these conditions demonstrate the necessary role played by dissimilarity in putting together similar components and segregating them from adjacent and surrounding elements. Dissimilarity and similarity can be considered as two complementary principles operating synergistically under the previous conditions. However, dissimilarity does not necessarily need similarity to be effective.

A point that is worth noting at this stage is related to the fact that dissimilarity does not emerge as promptly and strongly as similarities do. It is no coincidence that Wertheimer viewed and talked only about similarity. Apparently, the term “dissimilarity” could be assumed as another way to talk about similarity and, therefore, is useless to explain or describe something new. For example, to avoid any possible contradiction between the two terms, Vicario [16] suggested replacing similarity with “common quality”.

In line with our arguments, this phenomenal asymmetry showing mostly similarity requires explanation.

In accordance with the previous descriptions, if similarity highlights the inner object attributes and dissimilarity triggers the segregation, then the similarity is phenomenally expected to appear more saliently, at least in all the conditions where there is a large number of similar elements. Phenomenal exceptions are the cases illustrated in Figure 4, where the loneness of the red element tilts the balance in favor of the dissimilarity, showing this quality more strongly than similarity.

In the next section, the notion of visual information, considered as a mutual interaction between similarity and dissimilarity, is studied and extended to new unexplored qualities and complexities beyond the Gestalt principle of similarity.

## 3. On Dissimilarity in Reading

**Figure 10:**
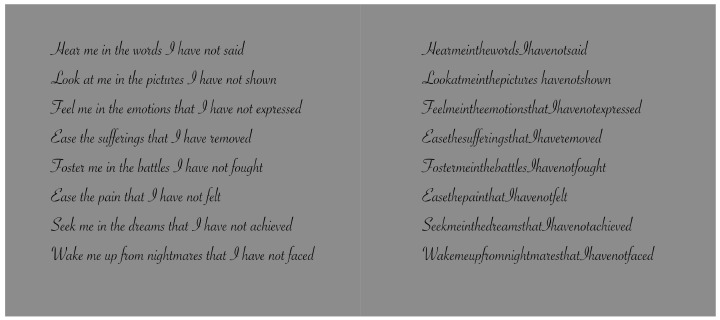
Written text with or without inter-word empty spaces.

To better understand the role of dissimilarity as a principle distinct from similarity, it is useful to create a temporal direction in the perception of elements that could reveal dissimilarity at first and then similarity. A possible appropriate candidate is to use written text, as illustrated in Figure 10 [22,23,24]. From one word to another, empty spaces help both perception and segmentation of the words to be read sequentially. By removing these gaps, the reading becomes hard and requires a lot more time. Moreover, the number of reading errors increases and the comprehension of the text worsens.

Gaps can be considered as special cases of dissimilarity. In some sense, they break off the continuity of juxtaposed letters, inducing a jump between words. They are equal to multiple lacks in homogeneity, discontinuities and, more generally, dissimilarities. Because of these attributes, they are used to segregate one word from another, or one object from another. By going through the two texts of Figure 10, the reader has a full and immediate demonstration of the role of discontinuity due to the gaps.

**Figure 11:**
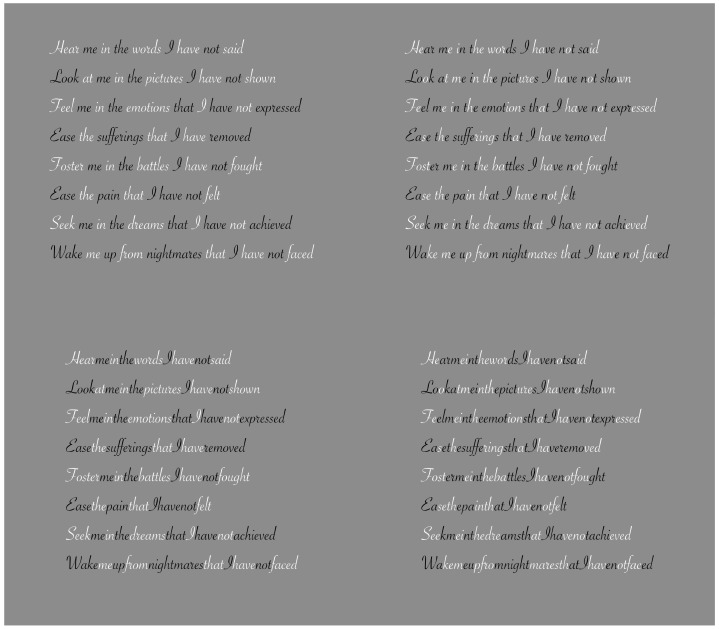
Contrast polarity used as a tool to improve or deteriorate reading with (**first row**) or without (**second row**) inter-word empty spaces.

The directional and sequential nature of reading from left to right is useful to unambiguously demonstrate the peculiar role of dissimilarity as a principle different from similarity. For this purpose, we now introduce the contrast polarity as a tool to improve or deteriorate reading. In the first row of Figure 11, empty spaces among words are preserved, but they have been removed in the second row. The reader can easily test the role of dissimilarity and, only later, that of similarity. Actually, the reading process under these conditions meets, at first, the dissimilarity and, secondly, the similarity.

**Figure 12:**
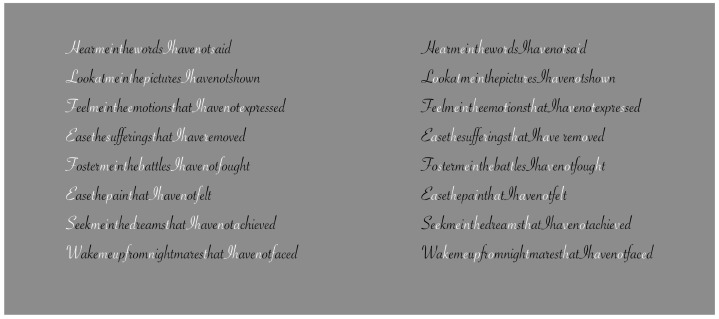
The role of dissimilarity in influencing reading when only a single letter is highlighted by the contrast polarity.

The role of dissimilarity is even more clearly demonstrated in Figure 12, where only a single letter is highlighted by the contrast polarity. Given the reading direction from left to right, both the improving and the deteriorating conditions support that the basic and unique role of dissimilarity is not reducible to similarity. Similar results are achieved by playing with colors instead of contrast polarity [22,23]. The kind of font does not significantly influence the basic outcomes.

**Figure 13:**
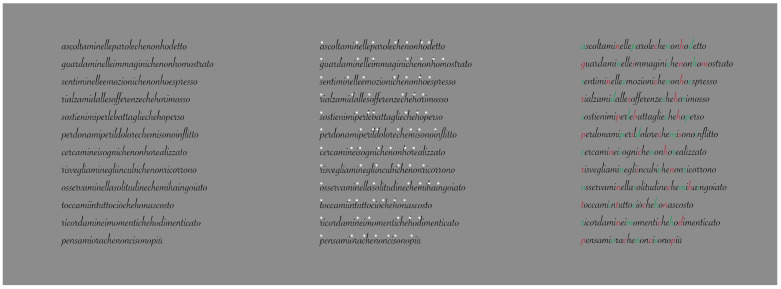
A single dot placed closer and over the first letter (**second condition**) and the first letter of each word chromatically alternated between red and green (**third condition**) improve the reading. Compare with the control (**first condition**).

Last but not least, in Figure 13—center, a stimulus (in Italian) used in our experiment is illustrated, where the dissimilar element is not one of the letters of the text but a dot placed closer and over the first letter. (This kind of dissimilarity will be further discussed and explored below.) In Figure 13—right, the first letter of each word is chromatically replaced and alternated with red and green. Figure 13—left is the control. The outcomes demonstrated the significant role of different kinds of dissimilarities in segregating one word from another, to improve or deteriorate reading, to influence the time spent reading, the number of errors and memory and comprehension of the text.

## 4. Dissimilarity to Create Something That Is Not There

**Figure 14:**
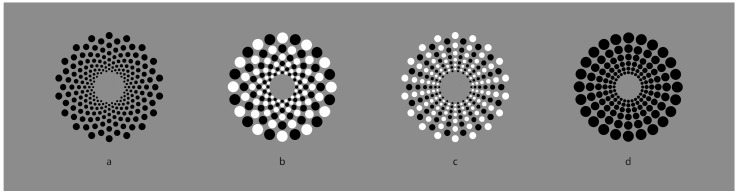
Spiraled arrangement of elements around a common center (**a**,**b**); radial grouping (**c**,**d**), although (**a**) is geometrically equal to (**c**) and (**b**) to (**d**).

If the task of dissimilarity is to segregate, then it can be used at will in many kinds of patterns to reveal a visual object that was not there at first. In this way, dissimilarity is a good player in a sort of visual hide-and-seek game. In Figure 14a,b, it is immediate to describe the two patterns as spiraled arrangements of elements around a common center, whose rays appear to turn in two opposite directions: clockwise and anti-clockwise. From periphery to the center, the size of the elements goes from the largest to the smallest. In Figure 14c,d, the only change is the element arrangement—now radial rather than spiraled.

Indeed, leaving out the reversed contrast, Figure 14a is geometrically the same as Figure 14c, while Figure 14b is equal to Figure 14d. These phenomenal outcomes demonstrate the role of similarity/dissimilarity in revealing what is absent or invisible when all the elements are black.

**Figure 15:**
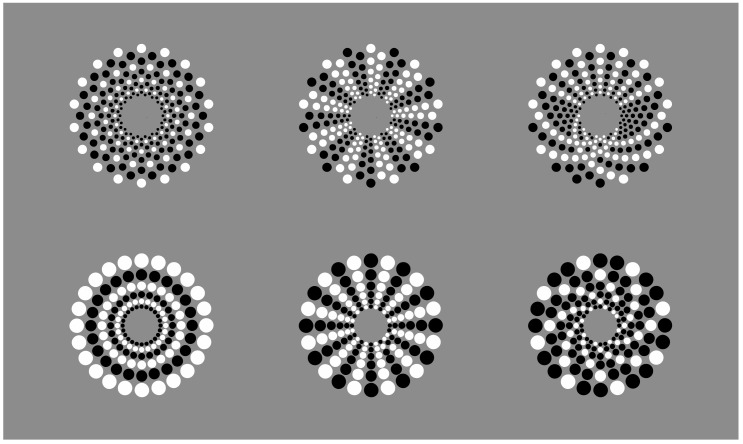
New perceptual grouping starting from Figure 14.

The two control patterns of Figure 14 (i.e., Figure 14a,d), can be rearranged by similarity/dissimilarity as illustrated, respectively, in the first and second rows of Figure 15. (It is worth noting the illusory concentric boundary contours perceived in the central condition of the second row, which is not further discussed here.) Under these conditions, in Wertheimer’s words, similarity of the contrast polarity wins against proximity. Other kinds of arrangements and shapes can be easily created (not illustrated).

**Figure 16:**
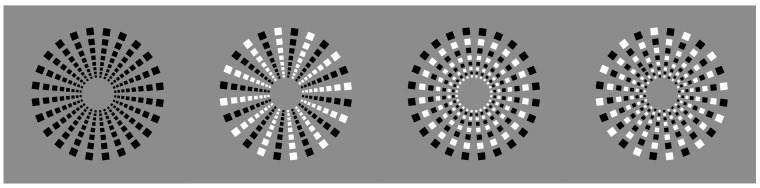
Grouping by proximity and similarity and a trapezoid distortion of each square mostly visible in the radial arrangements.

Similar variations in the arrangement of the elements can be achieved by replacing the circular elements with squares, as shown in Figure 16. The first two arrangements are radial with the first one due to proximity, the second to similarity. The third is concentric and the fourth is checker-boarded. A further phenomenon that is easily noticed is the trapezoid distortion of each square [25], mostly in the radial conditions, i.e., the first two. The longest side of each illusory trapezium is facing the center of the radial grouping, which enhances the effect that, in its turn, it is reduced in the concentric and checker-boarded arrangements. The direction and the eye movements imparted and following the arrangement of the checks increases or reduces this effect. Moreover, in the first two conditions, there are unique initial and ending points for one to begin or finish seeing. The trapezoid effect is stronger going from the periphery to the center of the rays than vice versa. Starting and ending points are absent in the other two conditions; as a consequence, the trapezoid illusion is reduced or absent.

In the radial arrangements, the dissimilarity involves two contiguous rays and, thus, it occurs circularly, while in the concentric grouping it refers to adjacent circles; hence, it goes radially. The opposite is true for the similarity.

**Figure 17:**
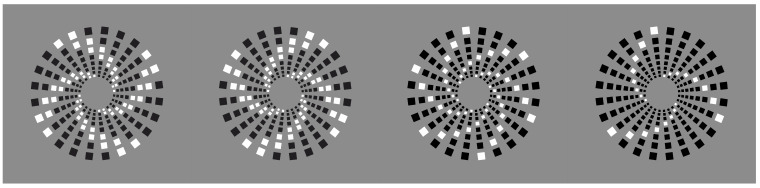
Spiral arrangements due to similarity/dissimilarity.

In Figure 17, the arrangement of the checks has been spiraled against the proximity principle. Within these patterns, two effects can be detected. The first is the spiraled outcome of both the white and black checks, except for the last pattern, where the spiral effect belongs only to the white checks, while the black ones follow the radial direction imparted by proximity. This double result might be considered as some kind of inconsistency or contradiction. The whole effect can also be described as an independent spiral place of or adjacent to some kind of tube going in depth. In this way, the contradiction is gone.

The second more interesting effect, mostly visible in the third and fourth patterns, is related to the way the shapes of the checks are perceived, i.e., closer to diamonds than to squares. Phenomenally, this kind of organization highlights the corners of the checks instead of the sides, as in the radial grouping. A similar result was previously described in Figure 8. This effect concerns the accentuation of different attributes within a shape as a result of grouping and dissimilarity. This will be examined in depth in the next section, since it represents a further complexity of perceptual organization not contemplated and that goes beyond the Gestalt principle of similarity.

## 5. Accentuation from Dissimilarity

A geometrical square is a regular quadrilateral with four equal sides and four equal angles. Hence, its main attributes are the sides and the angles. Phenomenally, they manifest different properties. Sides are flat and straight; angles are pointed and sharp. Squares and diamonds manifests univocally only one of the two properties. The square appears flat, showing sidedness, while the diamond is seen as sharp, emphasizing pointedness. In Figure 8c,d, we have seen that the whole matrix of dots can be perceived as a diamond or as a rotated square, depending on the position of the white dots.

Obviously, in the geometrical domain, there is no difference between a “diamond”, i.e., a square rotated by 45-degrees, and a “rotated square”, which is again a 45-degree rotated square. However, phenomenally, they are different since they highlight the two opposite attributes of pointedness and sidedness [25,26,27,28,29,30,31,32,33].

**Figure 18:**
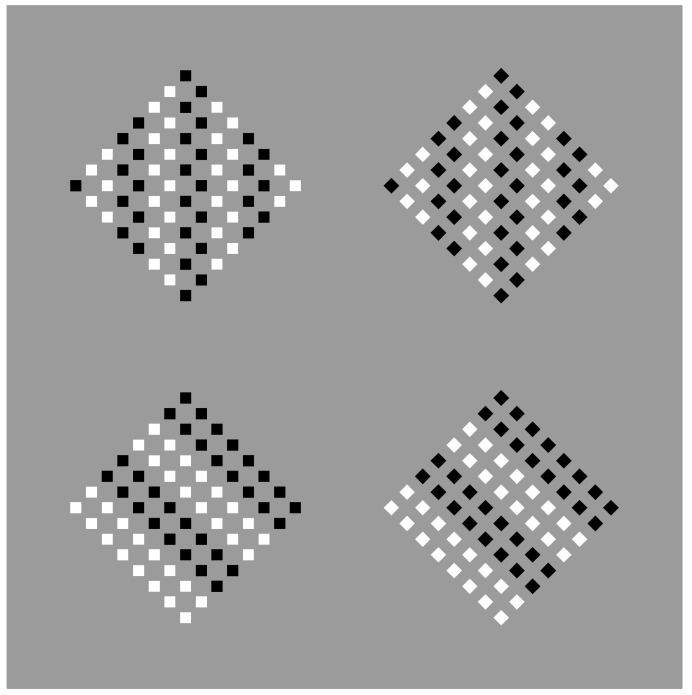
Squares perceived as squares (**1st**); diamond as diamonds (**2nd**); squares as diamonds (**3rd**) and diamonds as rotated squares (**4th**).

Figure 18 demonstrates these two attributes induced by the direction imparted by similarity/dissimilarity within the same geometrical patterns. Perhaps it would have been more appropriate to use phenomenally only the term “similarity”. However, we will demonstrate step by step that in conditions such as these ones, the main role is due to dissimilarity.

In Figure 18—first row, square elements appear as squares (left) and diamonds are perceived as diamonds (right). All of them are inset within a diamond-like shape, perceived, in turn, as a large diamond. This result is expected since the direction imparted by similarity runs and plays, accentuating the sides on the left and the angles on the right of both the inner checks and the wholes.

In Figure 18—second row, squares are perceived as diamonds (left) and diamonds as rotated squares (right). Likewise, the diamond wholes of both conditions are perceived as rotated squares. Now, the direction of the grouping accentuates the attribute that is opposite to the one corresponding to the geometrical shape, i.e., angles vs. sides and sides vs. angles.

**Figure 19:**
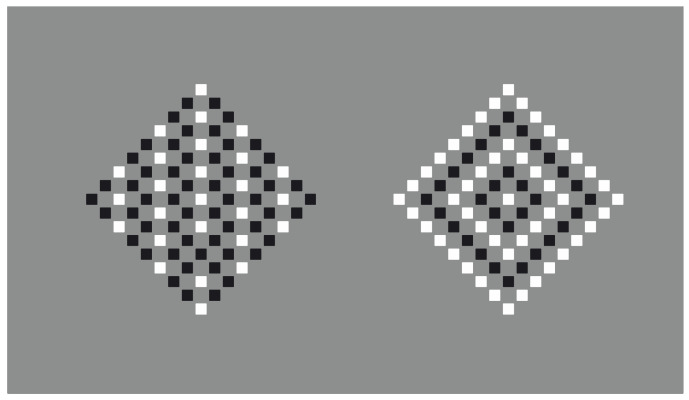
The same checks perceived as squares or diamonds.

**Figure 20:**
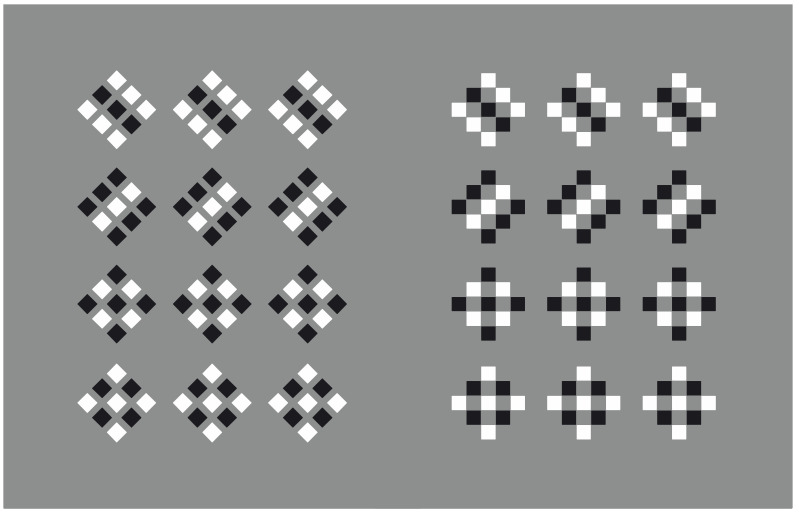
The same checks perceived as squares or diamonds.

**Figure 21:**
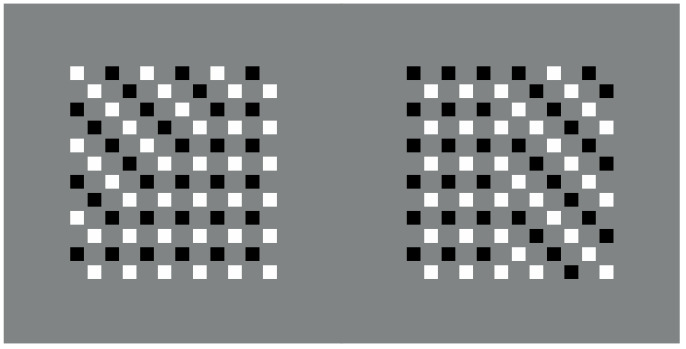
The same checks perceived as squares or diamonds.

**Figure 22:**
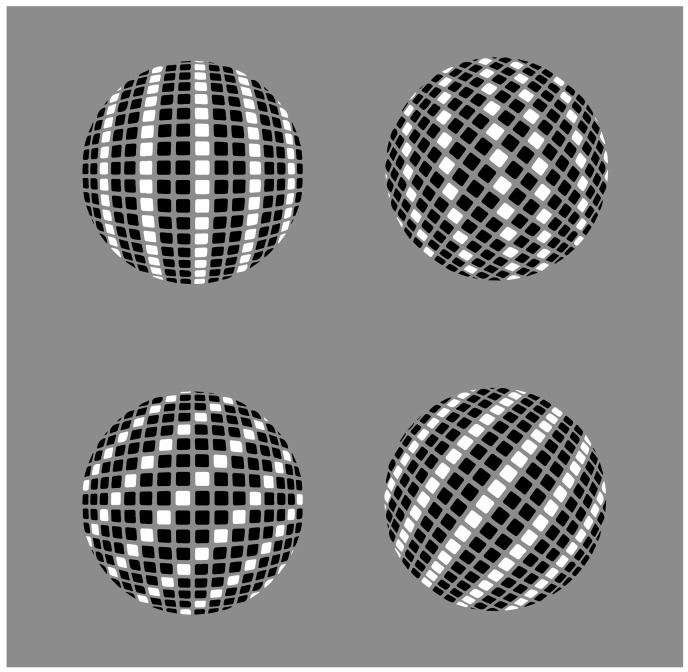
Squares and diamonds (first row) placed on the surface of a sphere perceived as diamonds and as rotated squares.

Other ways to make the checks appear as squares or diamonds because of the similarity/dissimilarity dynamics are illustrated in Figure 19, Figure 20 and Figure 21. In Figure 22, the same results occur with checks that appear to be placed on a spherical surface oriented as squares (first column) or diamonds (second column).

**Figure 23:**
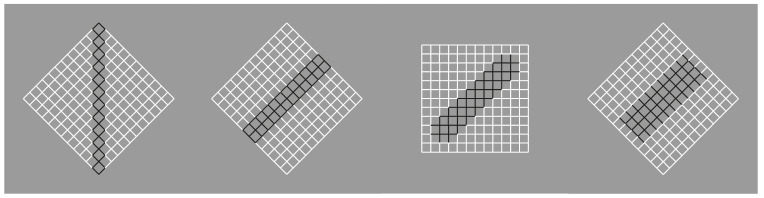
Crossed or parallel segments induced on a lattice by similarity/dissimilarity.

Pointedness (crossed lines) and sidedness (parallel lines) can be induced through the same dynamics on a lattice, as shown in Figure 23. Here, it should be noted that an alternative outcome could have been “a white lattice partially painted in black”. This is a supposed but not real outcome that ignores the role of dissimilarity, playing against the good continuation and the whole effect, and which breaks the lattice in two parts, as is clearly perceived. The strength of the dissimilarity against other Gestalt principles will be demonstrated in the next sections.

**Figure 24:**
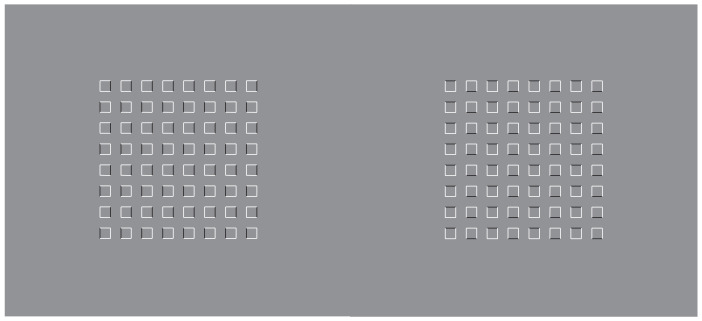
Lines of checks pointing and going alternately left and right (**left**) or up and down (**right**).

Figure 24 shows another effect due to the dissimilarity involving only the sidedness of squares, but in opposite directions. The results demonstrate lines of checks pointing and going alternately left and right (Figure 24—left) or up and down (Figure 24—right). A motion illusion can also be perceived [25].

**Figure 25:**
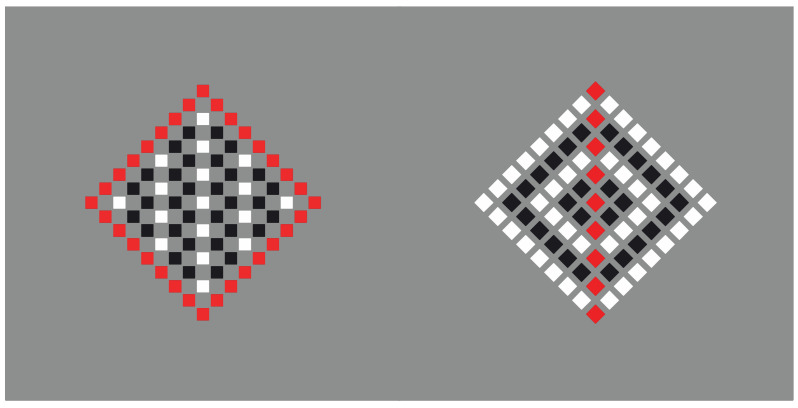
Different dissimilarities showing nested groups of diamonds or squares. Only the red dissimilarities are perceived as diamonds.

The play of dissimilarities can be multiplied by adding a new chromatic dissimilarity (Figure 25). Under these conditions, the different kinds of dissimilarities operate independently of each other, showing nested independent groups of diamonds or squares, one within the other. This set of nested dissimilarities is an issue not further deepened in this work.

**Figure 26:**
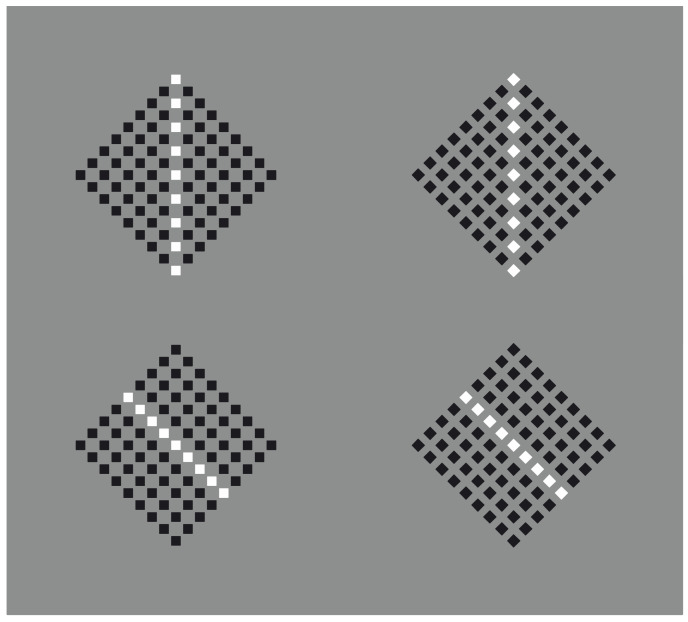
Dissimilar vertical or oblique alignment of similar elements perceived as squares vs. diamonds (**first column**) or as diamonds vs. rotated squares (**second column**).

In Figure 26, the alternated different similarities of Figure 18 are reduced to a dissimilar vertical or oblique alignment of similar elements. By focusing on the white components, the previous outcomes appear now even stronger on the white checks. It is worth noting that the same effects on the white elements spread on the black surrounding ones. The conditions of the first row are especially interesting if compared with those of the second row. In fact, even if the checks of the first pattern are aligned along the angles, the grouping along the vertical diagonal induces, within the check, a clear perception of sidedness (rotated square effect) rather than pointedness (diamond effect). The opposite occurs in the second pattern. This entails that the spreading of shape perception is likely stronger in the first row.

**Figure 27:**
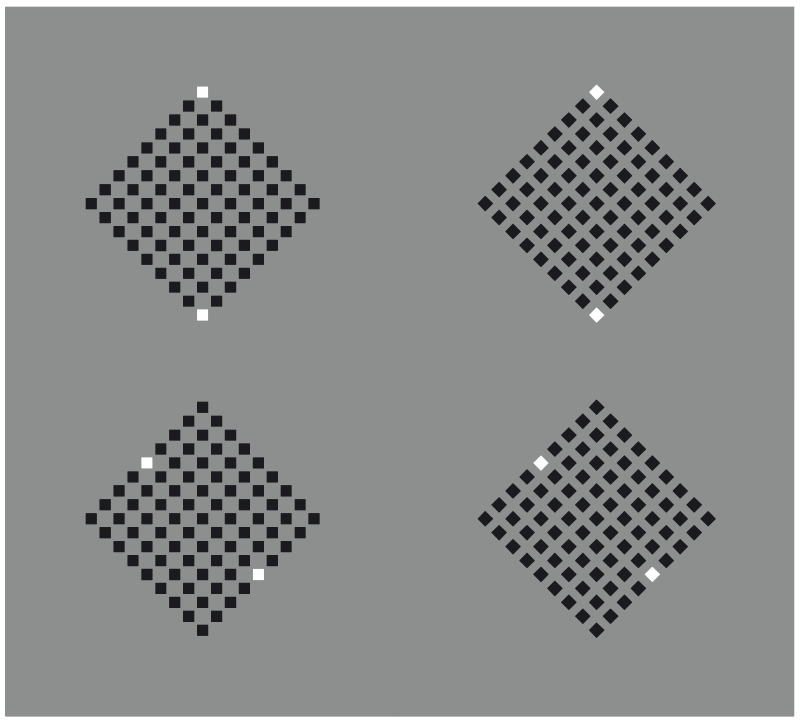
The same results of Figure 26 by reducing the vertical and oblique arrangements to only two checks placed at the antipodes.

**Figure 28:**
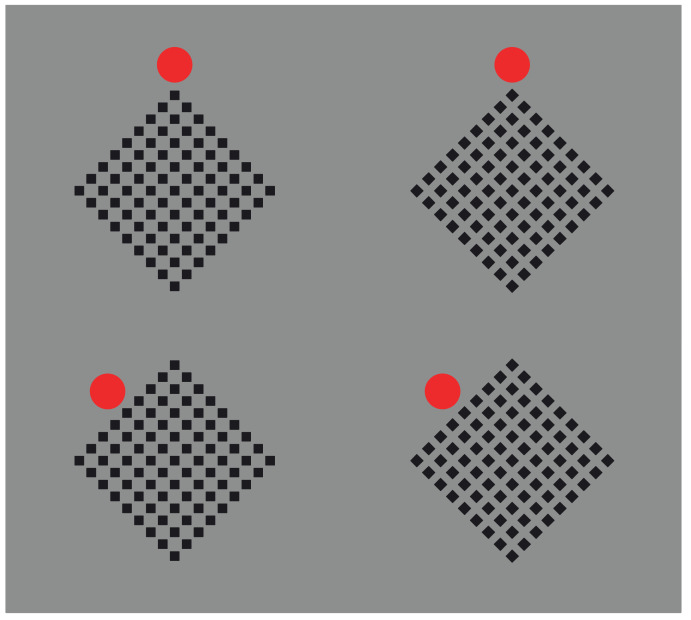
The red circles accentuate sides or angles within the checks, thus highlighting their appearance as diamonds or rotated squares.

The same results can be obtained by reducing the vertical and oblique arrangements to only two checks placed at the antipodes (Figure 27) or even to only one (not illustrated). However, the shape accentuation persists also when a totally dissimilar element is placed near the crucial components of the whole patterns (Figure 28).

**Figure 29:**
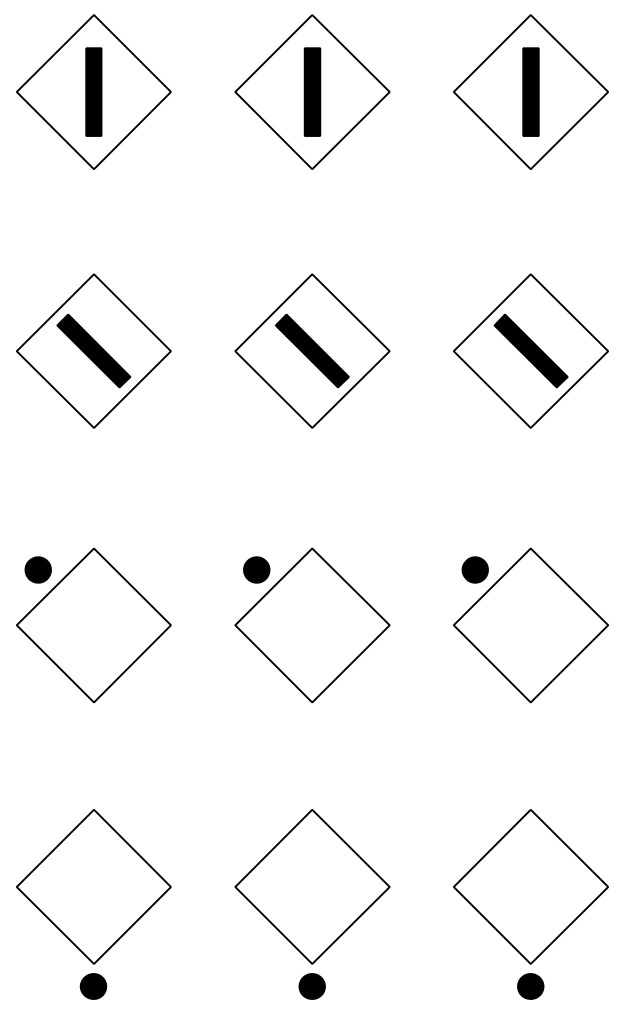
Accentuation of diamonds (**first and last rows**) and rotated squares (**second and third rows**) by dissimilarity.

Finally, the basic role of dissimilarity is fully demonstrated by reducing the pattern to single figures, as shown in Figure 29 [25,28,29]. Here, diamonds and rotated squares are perceived independently from similarity. The interaction between the dots and the geometrical shape is a demonstration of a perceptual organization and grouping in terms of accentuation, even against the configural orientational effect first studied by Attneave [34,35,36,37,38,39]. On the basis of configural orientation, in all the illustrated conditions, the results should be the same: diamonds.

## 6. Shape Distortions from Dissimilarity

**Figure 30:**
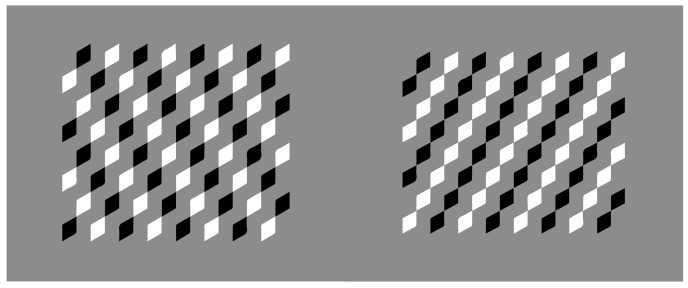
The rhombi on the left are perceived bigger and less tilted and less distorted than those on the right.

The interaction between similarity and dissimilarity among checks can elicit shape distortions such as those shown in Figure 30, where the same rhombic shapes are perceived as tilted mostly in the direction of the grouping, thus enhancing or reducing the tilt towards the longest diagonal. As a consequence, the rhombi of Figure 30—left are perceived as bigger, less tilted and less distorted than those of Figure 30—right.

**Figure 31:**
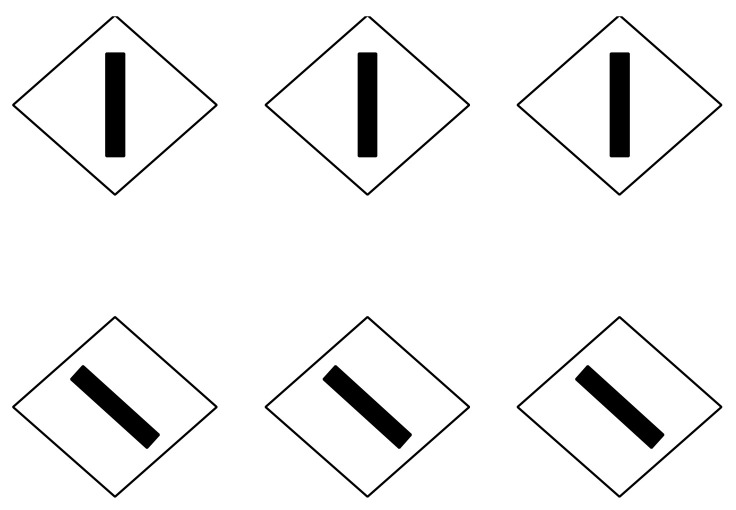
The rhombi of the two rows cannot be perceived as geometrically equal.

A similar result, due only to dissimilarity, is illustrated in Figure 31. Here, the rhombi belonging to the two rows cannot be perceived as geometrically equal, even if they truly are.

**Figure 32:**
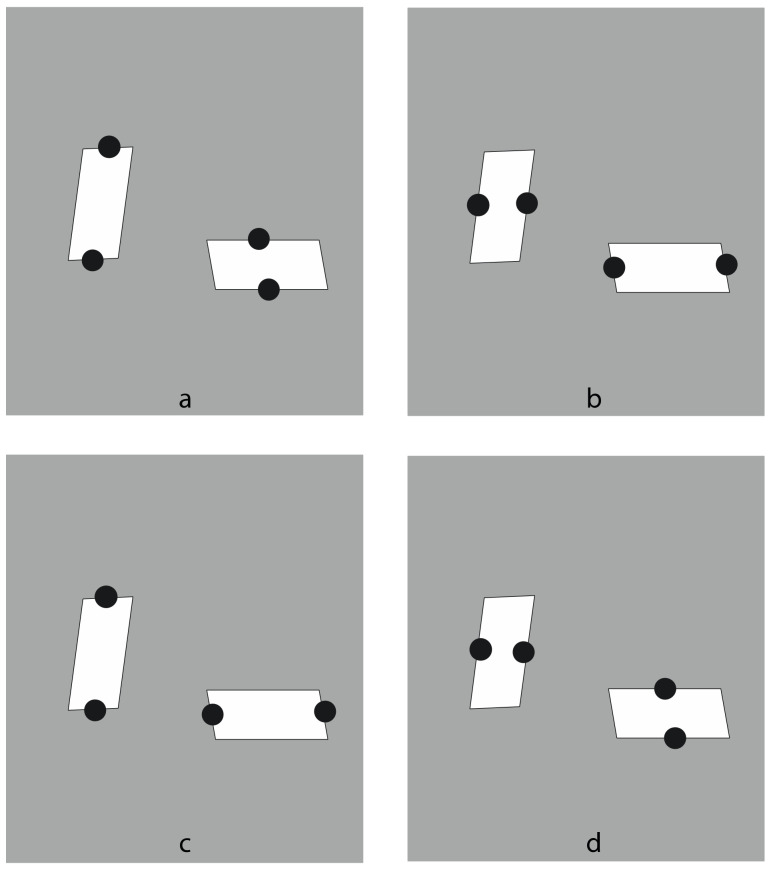
The same parallelogram vertically or horizontally oriented perceived as elongated (**a**,**c**; **b**,**c**) or enlarged (**b**,**d**; **a**,**d**) due to the dots placed on two opposite sides.

Last but not least, in Figure 32, we demonstrate several conditions where the same parallelogram vertically or horizontally oriented is seen elongated or enlarged due to the dots placed on opposite sides.

## 7. Illusions from Dissimilarity

**Figure 33:**
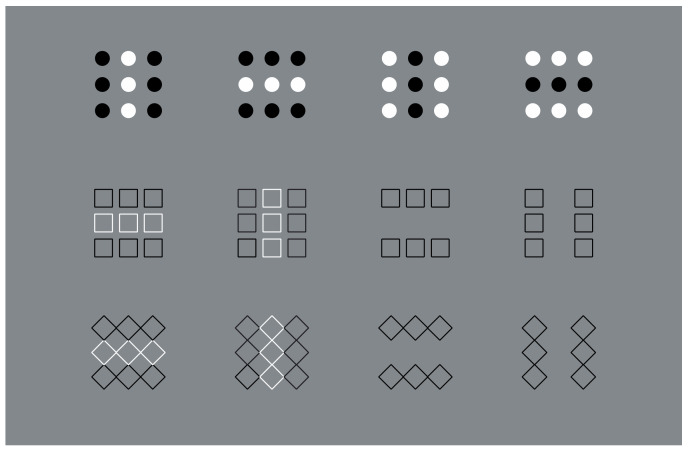
Despite that the shape of each pattern is a square, it appears as a vertical or as a horizontal rectangle following the direction of the grouping.

Dissimilarities among elements impart not only grouping, figure–ground segregations and accentuations of inner shape attributes and of new forms. They can also create shape illusions such as those demonstrated in Figure 33, where, despite the fact that the shape of each pattern is a square, it appears to be a vertical or a horizontal rectangle following the direction of the grouping. Therefore, for instance, vertical grouping delivers a vertical rectangle and a horizontal rectangle is delivered in the horizontal arrangement. This phenomenon involves not only the whole pattern but also the inner checks of the second and third row, which appear, likewise, as vertical or horizontal rectangles. It is noted that one of the dissimilarities used here is proximity, to be better described as a gap among checks.

**Figure 34:**
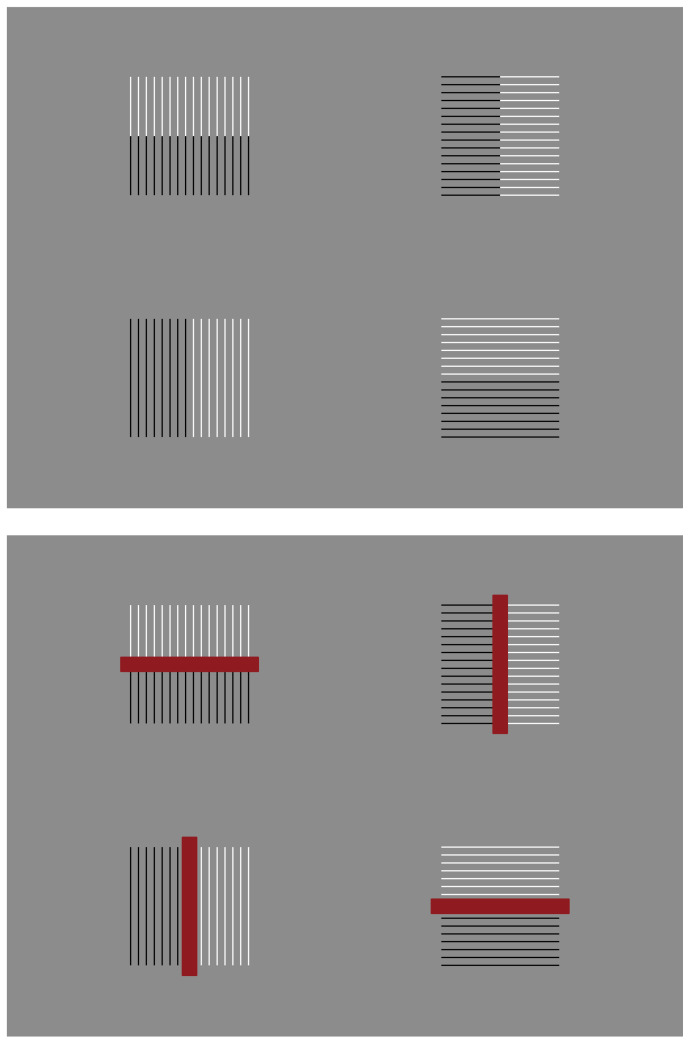
Similarities and dissimilarities interact to distort the whole squared bunch of parallel segments by making them slimmer or fatter.

Slimmer or increasingly fatter rectangles can be created by playing with dissimilarities, as illustrated in Figure 34—top, or by adding a further inner red dissimilarity to this stimuli (Figure 34—bottom). The whole shape of the bunch of parallel segments is the same square in all conditions.

**Figure 35:**
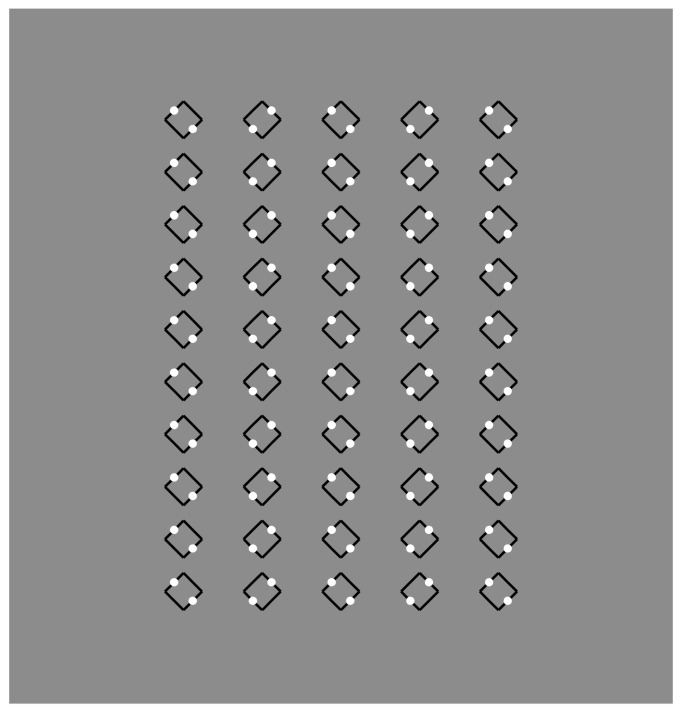
It is impossible to perceive the checks as the equal squares that they really are.

The rectangle illusion can be better perceived in Figure 35 as accentuated by the dissimilarity of the dots. Indeed, it is impossible to perceive the checks as equal squares, as they really are.

Phenomenally, the checks manifest a strong apparent sliding motion. This effect can be perceived clearly when the gaze follows the tip of a pen moving vertically or horizontally across the checks, but with attention and peripheral vision focused on the surrounding elements. While the pen is moving vertically the apparent motion is perceived horizontally and, vice versa, when the pen moves horizontally, the apparent motion occurs vertically.

## 8. Similarity to Reduce or Annul Illusions

**Figure 36:**
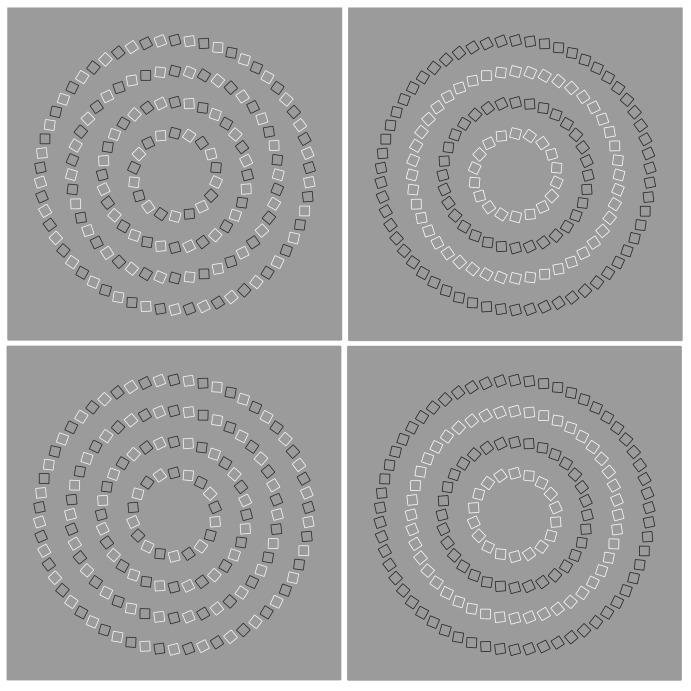
Dissimilarities among checks induce a double intertwined spiral on concentric annuli made of black and white squares (**first column, top**) and an equidistant spiral (**first column, bottom**). Similarity strongly reduces or annuls these effects (**second column**).

Similarity can reduce or annul illusions (Figure 36). In the first column of Figure 36—top, double intertwined spirals are perceived on a stimulus made up of concentric annuli composed of squares whose boundaries are alternately black and white. In the first column of Figure 36—bottom, a spiral with turns placed at the same distance is seen. The dissimilarities in the alternated squares seem to be responsible for most of the described illusory effects. In fact, by introducing the similarity on the squares of each ring, the phenomena just described are very weak or almost annulled.

## 9. Ungrouping from Dissimilarity

**Figure 37:**
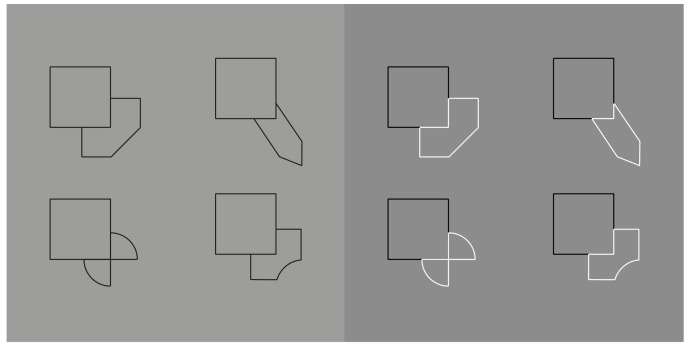
Dissimilarity ungroups and re-shapes the occluded object on the (**left**), which now appears as occluding a square (**right**).

Similarity and dissimilarity can also ungroup, for example, by playing against other principles and phenomena, such as good continuation and amodal completion. In Figure 37—left, four well-known stimuli, studied by Kanizsa [40,41], show the role of good continuation in defining the shape of the occluded object behind the square. The discontinuity of the T-junctions defines the phenomenon of amodal completion.

Now, by introducing the contrast polarity, the previous outcomes are easily ungrouped and re-shaped, as illustrated in Figure 37—right [6,25]. The occluded objects behind a square are now perceived as occluding the square and with shapes different from those perceived in Figure 37—left.

**Figure 38:**
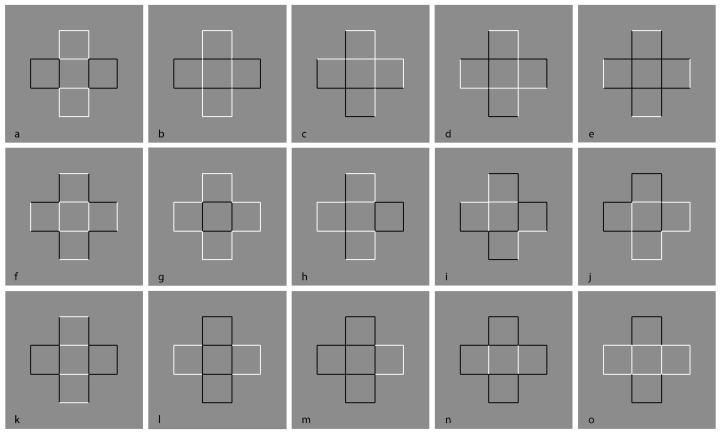
Grouping, ungrouping and reshaping the same element components through the reversed contrast.

Reversed contrast is likely the best tool to group and ungroup and, thus, reshape objects at will, even under a unique and very simple condition such as the one illustrated in Figure 38.

**Figure 39:**
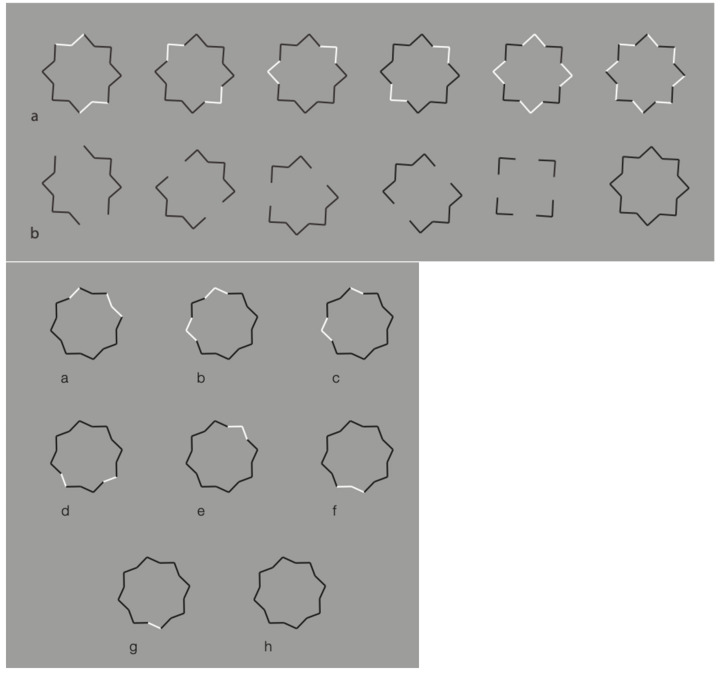
Ungrouping a star by making its sides and angles dissimilar.

However, the strongest and more-effective way to demonstrate the ungrouping power of dissimilarity due to reversed contrast is illustrated in Figure 39 where, by inverting the contrast of a few components of a star, we can obtain different shapes, such as those presented in Figure 39—second row. See also Figure 39—second set of figures.

**Figure 40:**
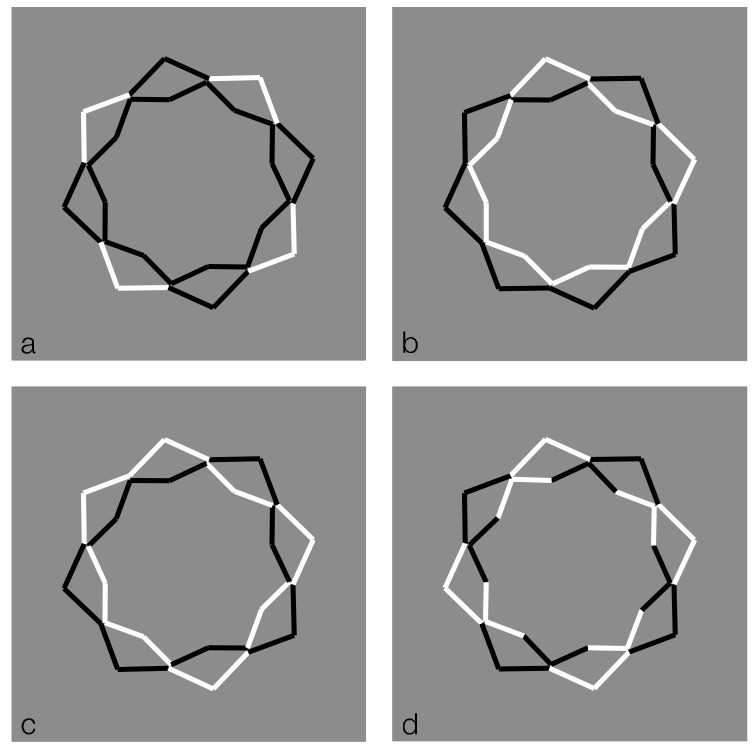
A double star, one inset on the other, ungrouped and puzzled by dissimilarity.

What makes these figures so powerful as a demonstration of the basic role of dissimilarity is the fact that this stimulus is a unique shape (a star), ruled by the closure Gestalt principle, not a texture and not a double star such as the ungrouped one in Figure 40.

## 10. Conclusions

Our work showed that the use of similarity alone is not sufficient to understand the dynamics of grouping fully and correctly and, more generally, that it is insufficient for understanding perceptual organization occurring both in the classical and, particularly, in the new phenomena here presented. The limits and incompleteness of the similarity principle revealed the basic, more general and stronger role of dissimilarity in perceptual grouping and figure—ground organization under a large variety of conditions.

The results suggest that both similarity and dissimilarity can deliver and solve real and useful issues in perceptual organization. The two attributes are not only the opposite ends of a continuum, but are primarily distinct factors of the formation of an object. Dissimilarity was shown as a basic principle of figure–ground segregation, as a tool able to create at will new groups and visual objects within patterns where they are totally invisible, as an attribute that is able to accentuate different shape components within the same object, as a way to distort shapes, to create visual illusions, but also to reduce or annul them and, finally, to decompose, ungroup and reshape single objects. The results demonstrated the necessity to introduce a principle of dissimilarity to complement the similarity already studied by Gestalt psychologists.

In more detail, dissimilarities have been shown to be responsible for creating segregation, distinction and separation between adjacent groups, and similarities to be responsible for putting the elements within each group together homogenously. Dissimilarities aim to divide boundaries and to emphasize discontinuities among regions of the visual field and similarities aim to enhance the inner homogeneity. In short, dissimilarities highlight and segregate the boundaries of an object, similarities reveal its inner surface qualities, such as the grain and the matter.

Metaphorically, we can think of two groups of people, for example, supporters to two opposing teams. The dynamics between dissimilarity and similarity highlight and magnify differences between the two groups of supporters and reduce or minimize differences among the members within each group. Analyzing the outcomes from the two different perspectives, dissimilarities are perceived in between the teams, eliciting a strong segregation and differentiation effect, and similarities are seen within the members of each group, inducing unification and uniformity.

The dissimilarity/similarity dynamics are very effective to explain complex human interactions such as, for instance, racism, extremism and political, sexual or age conflicts, etc. Moreover, a small dissimilarity in one state can result in large differences in a later state (see Figure 35, Figure 36, Figure 37, Figure 38 and Figure 39). A deep understanding of these dynamics cannot be based only on the similarity. Similarity is not sufficient. In short, dissimilarities create boundaries, barriers, differences, divisions and reactions to changes and surprises, while similarities produce uniformness, homogeneity and unity.

There is a further and more phenomenological demonstration of the distinction between the two factors. This is the spontaneous and independent use of the two terms in our language to describe perceptual organization, both from the point of view of what emerges as a distinct informative separation and differentiation and from the point of view of what appears as a uninformative uniformity and homogeneity.

In summary, we can phenomenally define dissimilarity as the information content related to all kinds of changes, breaks and discontinuities occurring along and within a potential homogeneity and continuity. In this sense, dissimilarity is gradient boosting, an abrupt break of uniformity producing “surprise”. More formally, dissimilarity can be associated with an unexpected discontinuity function occurring in the derivative of a gradient of visual attributes [25,42,43,44].

If an abrupt change is unlikely—has a low probability of occurring—and produces “surprise”, then we gained more information from this event than if the event had been something we were expecting. In essence, an unexpected event produces more information by changing our perception of the world. Dissimilarities can be considered as the information content of an event, and as such, it can be phenomenally measured.

There is a more general and intriguing point that can become a challenging source of discussion at this stage. It is related to the connections between dissimilarity, the notion of visual information and information in the sense of the theory of information.

In short, Shannon [45] quantified the amount of information in a signal, suggesting that it is the amount of unexpected data contained in the message. He considered information in that which is not random; therefore, information is not noise, but what unpredictably adds information. In a given set of possible data, the information of a message describing one of these data quantifies the symbols needed to encode the data in an optimal way. The term “optimal” means that the obtained code word will determine the datum unambiguously, isolating it from all others in the set, and will have minimal length, namely, a minimal number of symbols. Information theory is based on the measure of uncertainty in term of Shannon entropy (H) that is similar to the definition of entropy in thermodynamics according to Boltzmann’s principle. In simple terms, the entropy can be described as how long of a message (in bits) we need to convey the value of the stimulus.

The way Bateson [46] defined “information”, incapsulated in his famous motto: information is a difference that makes a difference, is also interesting for our purposes.

The notion of dissimilarity is likely the phenomenal attribute closest to the notion of information as previously described. Given outcomes within a probability space and the information content inherent in those outcomes, entropy denotes the expected information over the outcomes, i.e., the average information over all of the possible outcomes. Since dissimilarity is the information that describes the degree of surprise of an event, the entropy measures, on average, how surprised we are going to be by the outcomes.

Dissimilarities that make dissimilarities, paraphrasing Bateson, create and deliver objects and information reducing uncertainty and entropy. Therefore, according to information theory, entropy is, in our conditions, a measure of the degree of the uniformness of a variation within a pattern of stimuli. The higher the entropy, the closer the visual pattern is to having all of its outcomes being equally likely.

The attribute of dissimilarity developed in this work, although potentially appearing close to the notion of information, does not stand for it, even if it might be a good candidate. Deeper and more focused demonstrations are required, together with more crucial stimuli, to come closer to such a conclusion. At the moment, this is just an impromptu hint, a first look to be further explored.
